# Cell Proliferation and Apoptosis—Key Players in the Lung Aging Process

**DOI:** 10.3390/ijms25147867

**Published:** 2024-07-18

**Authors:** Jesús Ancer-Rodríguez, Yareth Gopar-Cuevas, Karol García-Aguilar, María-de-Lourdes Chávez-Briones, Ivett Miranda-Maldonado, Adriana Ancer-Arellano, Marta Ortega-Martínez, Gilberto Jaramillo-Rangel

**Affiliations:** Department of Pathology, School of Medicine, Autonomous University of Nuevo León, Monterrey 64460, Mexico; ancerrodriguezj@gmail.com (J.A.-R.); yareth.goparcu@uanl.edu.mx (Y.G.-C.); mdlourdes.chavezbrn@uanl.edu.mx (M.-d.-L.C.-B.); ivettmiranda77@gmail.com (I.M.-M.); adar7035@gmail.com (A.A.-A.); marta.ortegamrt@uanl.edu.mx (M.O.-M.)

**Keywords:** aging, cell turnover, cell proliferation, apoptosis, lung, lung diseases

## Abstract

Currently, the global lifespan has increased, resulting in a higher proportion of the population over 65 years. Changes that occur in the lung during aging increase the risk of developing acute and chronic lung diseases, such as acute respiratory distress syndrome, chronic obstructive pulmonary disease, idiopathic pulmonary fibrosis, and lung cancer. During normal tissue homeostasis, cell proliferation and apoptosis create a dynamic balance that constitutes the physiological cell turnover. In basal conditions, the lungs have a low rate of cell turnover compared to other organs. During aging, changes in the rate of cell turnover in the lung are observed. In this work, we review the literature that evaluates the role of molecules involved in cell proliferation and apoptosis in lung aging and in the development of age-related lung diseases. The list of molecules that regulate cell proliferation, apoptosis, or both processes in lung aging includes TNC, FOXM1, DNA-PKcs, MicroRNAs, BCL-W, BCL-XL, TCF21, p16, NOX4, NRF2, MDM4, RPIA, DHEA, and MMP28. However, despite the studies carried out to date, the complete signaling pathways that regulate cell turnover in lung aging are still unknown. More research is needed to understand the changes that lead to the development of age-related lung diseases.

## 1. Introduction

Aging is a physiological process that affects most living organisms. It can be defined as a time-dependent progressive functional deterioration that leads to a loss of physiological integrity, due to the accumulation of cellular damage, resulting in greater vulnerability to death [1].

In recent years, the global lifespan has increased and is expected to rise from 72.8 years in 2019 to 77.2 years in 2050, resulting in a higher proportion of the population over 65 years [2,3]. However, this has not been accompanied by an increase in people’s health. The healthy disease-free lifespan (healthspan) has not increased proportionally to the lifespan. From 2000 to 2019, the average lifespan increased by 6.5 years, compared to 5.4 years of the healthspan [4]. This poses a problem for healthcare institutions, since aging is considered the main risk factor for chronic non-communicable diseases like cancer and cardiovascular, neurodegenerative, and lung diseases [5,6].

## 2. Lung Aging

The lungs undergo functional and structural changes with age, even in the absence of disease. During the first 20 years of life, the lungs go through a phase of growth and maturation, until they reach their maximum function between 20 and 25 years; then, they undergo minimal changes until the age of 35, and subsequently the lung function begins to progressively decrease as age increases [6,7].

**Figure 1:**
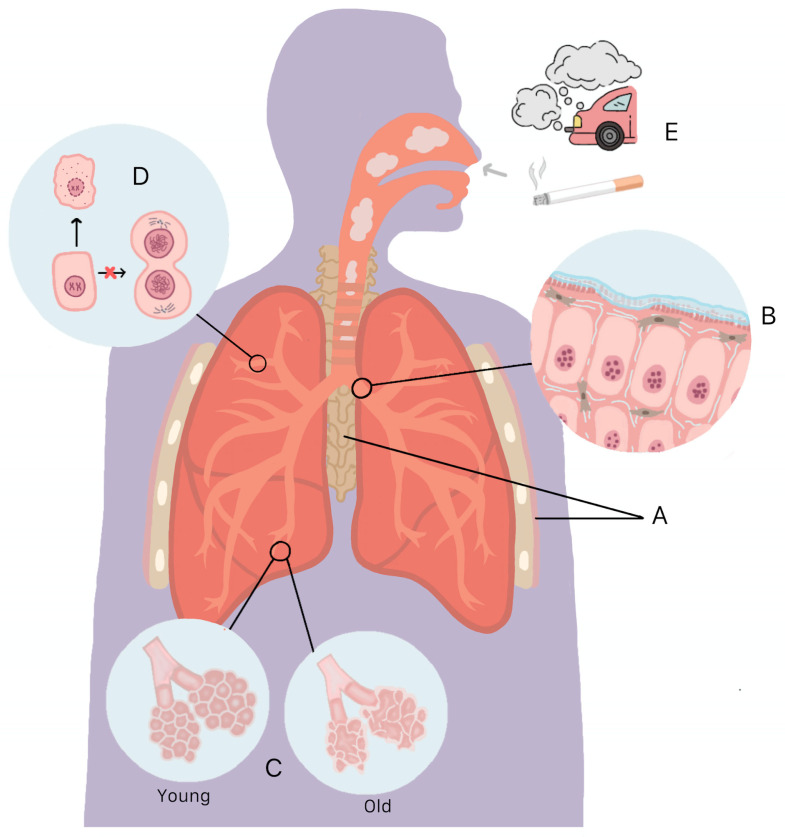
Anatomical and histological changes that occur in the lungs during aging. (**A**) Decreased respiratory muscle strength and deformities of the chest wall and thoracic spine. (**B**) Increased collagen production promotes changes in lung elasticity and reduced mucociliary clearance. (**C**) Increase in alveolar–capillary size and surface without destruction of the alveolar wall. (**D**) Telomere shortening and increased DNA damage promote cellular senescence. (**E**) Additionally, chronic exposure of the lungs to environmental pollutants promotes senescence and accelerated aging.

The decline in lung function with age is related to the structural changes that occur in the lung during the normal aging process. Structural changes can be at an anatomical or at a histological level. At the anatomical level, a decrease in the strength of the respiratory muscles occurs, as well as deformities of the chest wall and thoracic spine [8]. Among the histological changes is the deregulation of the extracellular matrix (ECM) due to greater collagen production, which promotes changes in lung elasticity, and an increase in alveolar size as well as in the alveolar–capillary surface without destruction of the wall of the alveoli [9,10,11]. Another histological change that occurs is a decrease in alveolar number and alveolar attachments [11,12]. It has also been reported that a reduction in mucociliary clearance, telomere shortening, as well as an increase in DNA damage and cellular senescence occur (Figure 1) [10,13].

Another important point to consider is that the lungs are constantly exposed to environmental pollutants such as cigarette smoke and vehicle exhaust gas. This promotes cellular senescence, accelerating lung aging [6,7,14].

Changes that occur in the lung during normal aging increase the risk of developing age-related acute and chronic lung diseases. Among the most frequent diseases are acute respiratory distress syndrome (ARDS), chronic obstructive pulmonary disease (COPD), idiopathic pulmonary fibrosis (IPF), and lung cancer [15,16]. Chronic lung diseases cause high rates of disability and mortality worldwide, posing a major global health problem [16].

### 2.1. Acute Respiratory Distress Syndrome (ARDS)

ARDS is a frequent cause of respiratory failure in critically ill patients, characterized by the acute appearance of non-cardiogenic pulmonary edema and hypoxemia. Patients with ARDS frequently present with diffuse alveolar damage and injury to both the alveolar epithelium and the pulmonary endothelium, resulting in the accumulation of protein-rich inflammatory edematous fluid in the alveolar space. It has a high mortality rate, between 30% and 40% [17,18].

ARDS can occur as a result of sepsis and pneumonia in people over 65 years of age, in whom it has an incidence up to 19 times higher and a mortality up to 10 times higher compared to young adults [19,20].

### 2.2. Chronic Obstructive Pulmonary Disease (COPD)

COPD is distinguished by a progressive lung function decline, leading to breathing difficulty. Patients may present features of chronic bronchitis and/or emphysema characterized by long-term airflow obstruction [21].

Airflow obstruction occurs due to obstructive changes in the peripheral airways and destructive changes in the respiratory bronchioles, alveolar ducts, and alveoli. The main symptoms include shortness of breath and cough with sputum production that usually worsens over time [21,22].

COPD is the third cause of death worldwide, causing more than 3 million deaths each year [23,24]. The incidence of COPD increases after the age of 45, and it is estimated that its global prevalence is around 3–6% among the population aged 40–49 years and 20–28% for those over 70 years of age [25,26].

### 2.3. Idiopathic Pulmonary Fibrosis (IPF)

IPF is a chronic and progressive disease of unknown origin. It affects the pulmonary interstitium and is characterized by the presence of fibrosis, inflammation, and destruction of the pulmonary architecture. Currently, this disease is incurable and has a survival rate of 3 to 5 years after diagnosis [27,28].

The disease is considered to develop due to the presence of constant microlesions in the alveolar epithelium during aging, which causes an interruption of epithelial–fibroblast communication, leading to the recruitment and activation of myofibroblasts that produce an ECM rich in collagen. Excessive accumulation of this matrix causes the alveoli to irreversibly collapse, resulting in reduced gas exchange and difficulty in breathing [29].

The average age of patients at the time of diagnosis of IPF is around 65–70 years, and although its incidence is low, its mortality rate is very high [30,31].

### 2.4. Lung Cancer

Lung cancer is the most common cancer in the world and the most common type in the elderly. About 30% of patients diagnosed with small-cell lung cancer and 50% of those diagnosed with non-small-cell lung cancer are over 70 years of age. However, smoking is still considered the main risk factor [32,33].

The greatest difficulty for elderly patients with lung cancer is the challenges that arise during their treatment. Aging produces physiological changes such as a decrease in bone marrow reserve, drug elimination, total body water, and lean body mass, as well as organ dysfunction. These changes increase the risk of death and the side effects of cancer treatment in older patients compared to younger ones [32,34].

## 3. Mechanisms Involved in Lung Aging

Cellular senescence is described as a state of irreversible cell cycle arrest; therefore, cell proliferation is affected. It occurs as a response against DNA damage to prevent the spread of damaged cells [26,35].

Senescent cells have been shown to accumulate with age and have therefore been established as a characteristic of aging and have been linked to a variety of age-associated diseases [36,37,38,39].

During normal tissue homeostasis, cell proliferation and apoptosis create a dynamic balance that constitutes the physiological cell turnover [40]. Accumulation of damage in cells increases the level of apoptosis and, in consequence, cell proliferation as a compensatory mechanism. However, an exaggerated cellular turnover accelerates cellular senescence [36]. Therefore, an increase in the number of senescent cells is inversely proportional to the proliferative and regenerative capacity [41].

Cell proliferation refers to the increase in the number of cells as a result of cell division through the progression of steps that constitute the cellular cycle [42]. On the other hand, apoptosis or “programmed cell death” is a genetically determined process by which a cell dies without spilling its intracellular contents into the surrounding environment; therefore, it does not activate the inflammatory response [43,44].

In basal conditions, the lungs have a very low rate of cellular turnover compared to other organs [45], and their low rate of stem cell division is enough to maintain the respiratory epithelium under normal conditions [6].

During normal aging, a decrease in the turnover capacity of lung epithelial cells is observed. Age is associated with an increase in the rate of apoptosis of bronchiolar epithelial cells (BECs), alveolar epithelial cells (AECs), and basal cells, as well as with a decrease in the rate of cellular proliferation of Clara cells [46,47,48,49]. Progenitor AECs type II (AEC-II) show increased senescence and metabolic alterations, which impairs their differentiation into AECs type I (AEC-I) [50,51,52].

Previously, our working group analyzed proliferation and apoptosis in the bronchiolar epithelium of mice of the CD1 strain at 2, 6, 12, 18, and 24 months of age. It was observed that as age increased, there was a decrease in cell proliferation and an increase in apoptosis, causing a decrease in cell turnover. Likewise, it was observed that the area and height of BECs decreased with the increasing age of the subjects [49].

In this work, we review the literature that describes the changes that occur in proliferation and apoptosis in the lung during the aging process to better understand the modifications that promote susceptibility to age-related lung diseases.

## 4. Cell Proliferation and Apoptosis in Normal Lung Development

Cell proliferation and apoptosis play an important role in normal growth and remodeling of the lung throughout life [53,54]. The expression of different molecules regulates these processes from the neonatal stage to the adult stage.

### 4.1. Fas/FasL System

The loss of AEC-II in the lungs during the post-canalicular phase is thought to occur through the terminal differentiation of AEC-II into AEC-I, but recent observations suggest that apoptosis may also play an important role in the perinatal homeostasis of AEC-II [54]. Previously, a correlation was observed between increased apoptosis of AEC-II and positive regulation of the pulmonary expression of Fas ligand (FasL) in fetal rabbit lungs [55].

The binding of FasL to its receptor Fas causes the activation of apoptosis [56,57]. Fas-FasL-mediated apoptosis is important for the maintenance of immune homeostasis. During a physiological immune response, apoptosis helps in the elimination of self-reactive lymphocytes, limiting the tissue damage caused by immune responses [58].

De Paepe et al. determined the spatio-temporal induction of lung apoptosis and the expression of the Fas/FasL system from embryonic day 17 (E17) to postnatal day 5 (P5) in mice of the C57BL/6J strain. During the evaluation of apoptosis, they determined the number of apoptotic nuclei per total number of nuclei (apoptotic index). The apoptotic index of AEC-II was 0.1% at E17, 1.5% at P1–P3, and 0.3% at P5. In the P1–P3 period, the highest proportion of apoptotic cells was observed; this time coincided with a 14-fold increase in Fas protein and mRNA levels and a 3-fold increase in FasL protein levels in AEC-II [59]. These findings indicate that the Fas/FasL system is a critical modulator of prenatal AEC-II apoptosis.

### 4.2. Erythropoietin Receptor (EPO-R)

Erythropoietin (EPO) was initially described as an essential cytokine for erythropoiesis. Recently, it has been discovered that it has other functions such as cytoprotection against pathological processes. EPO and its receptor (EPO-R) are expressed in various tissues, including the lung. EPO-R has been reported to be expressed in almost all cells of the pulmonary tissue, including BECs and AEC-II [60].

Foster et al. evaluated EPO-R expression during postnatal lung maturation at 3 months of age and in the mature lung at 12 months of age in normal dog tissue; they also evaluated EPO-R expression during compensatory lung growth at 3 weeks and 10 months after pneumonectomy (PNX) of the right lung. As a result, they found that EPO-R was expressed in a higher proportion during normal postnatal lung maturation and in the early stage (3 weeks after PNX) of injury repair, compared to the mature lung and the late stage (10 months after PNX) of injury repair. These results indicate that a decrease in EPO-R expression occurs with increasing age. Additionally, this receptor is necessary for lung repair after injury. Therefore, the decrease in regenerative capacity dependent on proliferation and apoptosis in the lung is related to the decrease in EPO-R expression with increasing age [61,62].

### 4.3. Tenascin-C (TNC)

Tenascin-C (TNC) is an ECM protein contributing to gastrulation and carcinogenesis [63]. Its expression significantly increases during alveolarization from postnatal day 4 to 21, but its expression is markedly reduced afterward [64].

Mund et al. investigated the impact of TNC deficiency on lung development during the formation and maturation of alveolar septa in the postnatal stage. They evaluated the lungs of the *Tnc*-null mouse strain “Tnc tm1Ref” between postnatal days 2 (P2) and 86 (P86). They observed that the septa of the alveolar space were atypically thickened with an accumulation of capillaries and connective tissue and also observed an increase in the number of cells in the lungs lacking TNC on day P7. Cell proliferation was significantly higher on days P4 and P6, and the total number of lung cells increased on days P10 and P14 in TNC-deficient mice compared with the control group. Finally, they observed that TNC-deficient lungs showed an increase in apoptotic cells at postnatal day 10. These findings demonstrate that TNC contributes to the formation of new alveolar septa by regulating cell proliferation and apoptosis during postnatal lung development [65].

### 4.4. Receptor for Advanced Glycation End Products (RAGE)

The receptor for advanced glycation end products (RAGE) is a member of a superfamily of cell surface immunoglobulins. This receptor is expressed at high concentration during embryonic development in various tissues and postnatal alveolar remodeling [66,67]. Afterwards, its expression levels decrease in most adult tissues, except the lungs [68]. In normal adult lung tissue, RAGE has been reported to have selective expression in the basolateral membrane of AEC-I [69]. The continuation of RAGE expression in the adult lung suggests a possible role in lung homeostasis, because RAGE regulates many cellular processes like cell proliferation and migration, inflammation, and apoptosis [70].

Fineschi et al. evaluated the overexpression of human *Rage* in mice of the C57Bl/6J strain during lung development at 4, 8, and 20 days after birth. Heterozygous overexpression of *Rage* during lung development caused alterations in the development of secondary alveolar septa, causing a decrease in the number of alveoli and an increase in air space, due to an increase in apoptosis and a decrease in proliferation in alveolar cells. Interestingly, when *Rage* was homozygously overexpressed, in addition to the presence of the alveolar alterations described previously, a thickening of the alveolar interstitium, with hypercellular septa and abnormal vascular development, was observed due to a mild medial thickening and abnormalities in the architecture of elastic fibers; these histological alterations are also observed in human bronchopulmonary dysplasia [71].

Go et al. analyzed the levels of soluble RAGE (sRAGE) in umbilical cord blood obtained from newborns who were less than 32 weeks of gestational age and who were mechanically ventilated or oxygenated. The serum sRAGE concentration was significantly lower in premature neonates compared to healthy neonates. In addition, among premature newborns, the blood levels of sRAGE were significantly lower in newborns with bronchopulmonary dysplasia compared to those without this pathology [72]. These results correspond with the previously described findings because sRAGE causes the competitive inhibition of the activation of RAGE found in the cell membrane; this indicates that when the sRAGE levels are decreased, greater RAGE activation occurs in the cell membrane [73].

In contrast, when *Rage* is eliminated in mice that are allowed to age normally, at 48 weeks of age, the subjects develop pulmonary fibrosis [74].

To date, the exact role of RAGE in pulmonary homeostasis is unknown. However, the regulation of its expression levels is important, because an excess in its levels during lung development alters the normal histology of the alveoli, and its absence triggers pulmonary fibrosis during aging.

**Figure 2:**
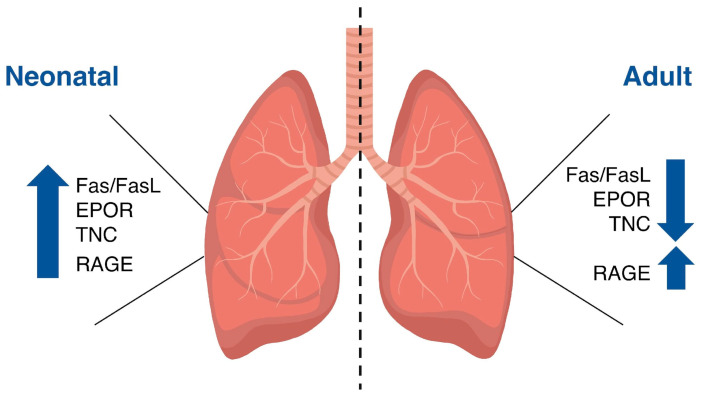
Expression pattern of molecules that regulate proliferation and apoptosis during normal lung development. As age increases, the expression levels of the Fas/FasL system, erythropoietin receptor (EPOR), and tenascin-C (TNC) decrease, while the levels of the receptor for advanced glycation end products (RAGE) remain elevated.

Figure 2 summarizes the changes that occur in the expression pattern of the molecules previously described during normal lung development.

## 5. Role of Cell Proliferation and Apoptosis in Lung Aging

### 5.1. Cell Proliferation

#### 5.1.1. Tenascin-C (TNC)

Tenascin-C (TNC) is an ECM protein highly expressed in the embryonic period during organogenesis [64]. In healthy adult lungs, this protein is found at low levels in the smooth muscle and basal cells of the airways [75]. However, its expression increases during tissue repair because it contributes to the regulation of cell proliferation, growth, and migration [76,77].

To analyze the role of TNC during aging, Gremlich et al. evaluated lung structure and physiology in 18-month-old *Tnc* knockout (*Tnc* KO) mice. They reported that at 18 months of age, *Tnc* KO mice showed greater lung aging compared to the control group because they had greater lung volume, parenchymal volume, total airspace volume, and septal surface area and presented an increase in total lung collagen, without alteration in the lung function. Also, they observed that TNC deficiency caused an increase in cell proliferation in the lung parenchyma. Interestingly, no differences were observed in the levels of apoptosis or cellular senescence between the groups analyzed, indicating that the observed changes occurred due to increased tissue production rather than a decrease in cellular destruction [78]. These results demonstrate the importance of TNC in delaying the changes that occur during lung aging.

#### 5.1.2. Forkhead Box M1 (FOXM1)

The forkhead box M1 (FOXM1) protein is a transcriptional regulator of cell cycle-related genes [79]. Smirnov et al. demonstrated that FOXM1 contributes to the maintenance of a high proliferative potential in keratinocytes, while its expression decreases during differentiation, as well as during replication-induced cellular senescence [80]. On the other hand, Ribeiro et al. demonstrated that the induction of FOXM1 expression in naturally aged C57BL6 strain mice results in delayed aging and prolonged lifespan [81].

FOXM1 also plays an important role in vascular repair. In *Foxm1* knockout mice, specifically in endothelial cells (ECs) after inflammatory injury, pulmonary EC proliferation and endothelial barrier recovery were found to be defective [82,83]. Endothelial injury is a hallmark of ARDS. The common causes of ARDS include sepsis and pneumonia [84,85]. Age is a major risk factor for the development of ARDS and death from it.

Huang et al. evaluated the effect of aging on FOXM1-dependent vascular repair and endothelial regeneration after inflammatory lung injury in mice of the strain C57/BL6. Following sepsis-induced vascular injury, they reported that endothelial regeneration was facilitated by the proliferation of lung-resident ECs in young mice (3–5 months old). Endothelial regeneration was impaired in aged mice (19–21 months old). Therefore, as individuals age, their ability to regenerate endothelial cells is impacted, resulting in long-lasting lung inflammation [84]. In aged mice after lung injury, FOXM1 was not expressed; therefore, its target genes such as cell division cycle 25C (*Cdc25c*) and cyclin A2 (*Ccna2*), which are essential for cell proliferation, were not expressed either. To confirm these results, they performed the induction of *FoxM1* expression in lung ECs in aged mice. FOXM1 increased cell proliferation, which caused the activation of pulmonary endothelial regeneration and promoted cell survival after lung injury [86]. Taken together, these data indicate that FOXM1 is necessary for pulmonary endothelial regeneration by regulating cell proliferation; therefore, inducing its expression could prevent the development of ARDS.

#### 5.1.3. DNA Protein Kinase Catalytic Subunit (DNA-PKcs)

The DNA-dependent protein kinase catalytic subunit (DNA-PKcs) is a member of the phosphatidylinositol 3-kinase (PI3K) family of kinases. It is part of the DNAPK complex, which is formed by the Ku70/Ku80 heterodimer and the central catalytic holoenzyme DNA-PKcs. The complex is activated upon binding of Ku70/Ku80 and DNA. Activation induces the autophosphorylation of DNA-PKcs as well as the phosphorylation of several other proteins, culminating in cell cycle checkpoint activation and DNA repair. Therefore, it is considered a master regulator of the DNA damage response [87,88].

DNA damage and a loss of the DNA damage response and repair pathways have been reported to occur in pulmonary fibrosis [89,90]. Habiel et al. analyzed the role of DNA-PKcs in IPF. They obtained biopsies and primary lung fibroblasts from patients with IPF and control subjects without IPF; the age range of the patients was 57–71 years. During the analysis, it was observed that the lung tissue of the IPF patients showed a decrease in the expression of DNA-PKcs, compared to that of the control group [91].

To identify the mechanisms by which DNA-PKcs deficiency participates in the development of IPF, they performed the inhibition of DNA-PKcs activity using the inhibitor Nu7441 in primary fibroblasts isolated from the lung of the control subjects. Inhibition of DNA-PKcs activity caused the proliferation of SSEA4+ mesenchymal progenitor cells, which were previously identified as mediators of IPF because they have the capacity to form fibrotic lesions in vivo [6]. An increase in myofibroblasts, inflammatory markers, and markers associated with cellular senescence was also observed [91,92].

#### 5.1.4. MicroRNAs

MicroRNAs are small (19–25 nucleotides) non-coding RNAs found in animals, plants, and some viruses. They negatively regulate gene expression at the messenger RNA (mRNA) level, through their binding to complementary sequences in the 3′ untranslated regions (3′ UTR) of their target mRNAs. MicroRNAs regulate a large number of cellular functions, including cell proliferation, growth, differentiation, apoptosis, and senescence [93,94].

Markopoulos et al. compared the expression profile of microRNAs between young human lung fibroblasts and fibroblasts in replicative senescence, obtained from the HFL1 cell line. They identified 15 microRNAs that were upregulated in senescent cells, i.e., let-7d-5p, let-7e-5p, miR23a-3p, miR-34a-5p, miR-122-5p, miR-125a-3p, miR-125a-5p, miR125b-5p, miR-181a-5p, miR-221-3p, miR-222-3p, miR-503-5p, miR574-3p, miR-574-5p, and miR-4454. The positive regulation of these micro-RNAs was associated with the arrest of the cell cycle in the G1/S phase, causing a decrease in cell proliferation [95].

Maes et al. evaluated the expression profile of microRNAs, in comparison to young human fibroblasts of the WI-38 cell line, in the following cells: (1) cells with reversible cell cycle arrest induced by nutrient depletion; (2) cells in replicative senescence; (3) cells in premature senescence induced by hydrogen peroxide (H_2_O_2_). The three groups analyzed showed an increase in the expression of miR-10b, miR-34a, miR-373, miR-377, miR-609, miR-624, miR-633, miR-638, and miR-663. The most robust upregulation of miR-638 and miR-663 was observed in the replicative senescence group, while that of miR-10b, miR-34a, miR-373, miR-377, miR-609, miR-624, and miR-633 was observed in the premature senescence group. The increase in the expression of these microRNAs was related to decreased cell proliferation [96].

In both studies, a relationship was observed between the positive expression of microRNAs related to cellular senescence and cell cycle arrest. Variations in the microRNA profile may depend on the cell line used. miR-34a was the only one with increased levels in both studies.

Cui et al. evaluated the effect of miR-34a on IPF. They characterized the expression of miR-34a in the lungs of young (10-week-old) and aged (20-month-old) mice. They found that, compared to young mice, miR-34a expression was increased in the lung epithelial cells of aged mice. An increase in miR-34a levels was also observed in aged mice treated with bleomycin to induce pulmonary fibrosis. To further evaluate the role of miR-34a in lung fibrosis, they used aged *miR-34a* knockout mice (20 months old) and the bleomycin lung fibrosis model. Wildtype mice showed a decrease in cell proliferation and a significant increase in collagen deposition compared to miR-34a knockout mice; therefore, deletion of miR-34a protected aged mice from lung fibrosis induced by bleomycin. This suggests that miR-34 plays a role in promoting the development of pulmonary fibrosis in aging [97].

### 5.2. Apoptosis

#### 5.2.1. BCL-W and BCL-XL

As previously mentioned, cellular senescence is a protective mechanism in response to cellular damage [36]. However, during aging, there is an increase in and an accumulation of senescent cells, which leads to the development of lung age-related pathologies [38,39]. Therefore, it is important to identify the changes that generate resistance to the elimination by apoptosis of senescent cells.

Yosef et al. evaluated resistance to apoptosis in cells undergoing senescence induced by three different mechanisms, as follows: (1) DNA damage-induced senescence (DIS); (2) replicative senescence (RS); (3) oncogene-induced senescence (OIS). For this, they used the IMR-90 primary fibroblast cell line. They observed that senescent cells (DIS, RS, and OIS) were more resistant to apoptosis induced by intrinsic and extrinsic pathways than non-senescent cells. This resistance occurred due to the upregulation of the antiapoptotic proteins BCL-W and BCL-XL in senescent cells [98].

Once the mechanism by which senescent cells resist apoptosis was established, Yosef et al. tested a treatment with ABT-737, which is an inhibitor of BCL-W and BCL-XL, in mice with lung damage and senescence induced by ionizing radiation (DNA DIS). Seven days post irradiation, the mice received ABT-737 for 2 days. The lungs were then analyzed 1 day later. As a result, the authors observed a significant decrease in the number of senescent cells, accompanied by an increase in activated caspase-3 after treatment with ABT-737, suggesting an increase of apoptosis in the DNA DIS lung. These findings suggest that senescent cells can be eliminated in vivo through inhibition of BCL-W and BCL-XL, as a strategy to prevent or treat lung age-related diseases [98].

#### 5.2.2. Transcription Factor 21 (TCF21) and p16

Telomerase is the enzyme responsible for maintaining telomere length by adding repetitive guanine-rich sequences [99]; this delays senescence and induces the immortalization of cells, as a protective mechanism against aging. Therefore, inhibition of telomerase can induce senescence or apoptosis [100]. The cellular mechanisms that determine the path that cells will take between senescence and apoptosis are unknown.

Selvam et al. evaluated the effect of silencing sphingosine kinase 2 (SPHK2), which helps in stabilizing telomerase for its proper functioning. When *Sphk2* was knockdown in p16-deficient lung cancer cells, induction of caspase-3-dependent apoptosis occurred through the activation of transcription factor 21 (TCF21). In contrast, when *Sphk2* was knockdown in non-cancerous cells, the cells became senescent, due to p16 activation. These data demonstrate that p16 abundance induces senescence and prevents apoptosis in response to telomere damage. In contrast, in the absence of p16 (as occurs in cancer cells), apoptosis is induced, and senescence is prevented as a response to prevent tumor growth [101].

#### 5.2.3. NADPH Oxidase-4 (NOX4) and NFE2-Related Factor 2 (NRF2)

NADPH oxidases (NOX) catalyze the reduction of oxygen to produce reactive oxygen species (ROS). This group of enzymes includes seven members: NOX1–5 and DUOX1–2. NADPH oxidase 4 (NOX4) is the only isoform that produces high levels of H_2_O_2_ [102]. On the other hand, NFE2-related factor 2 (NRF2) controls the expression of a variety of genes dependent on antioxidant response elements, acting as an antioxidant defense system [103]. An imbalance between the excessive generation of ROS and antioxidant defenses affects cellular homeostasis, promoting the development of different pathologies.

Hecker et al. evaluated NOX4 expression in fibroblasts isolated from the lungs of patients diagnosed with IPF. They observed higher concentration of NOX4 in fibroblasts obtained from patients with IPF than in control subjects without IPF. To evaluate the role of NOX4 in fibroblasts, they used GKT137831, a NOX1/4 inhibitor. Fibroblasts treated with GKT137831 showed a decrease in senescence-associated β-galactosidase (SA-βgal) activity, suggesting that NOX4 contributes to the cellular senescence of fibroblasts isolated from IPF. They also evaluated the role of NOX4 in apoptosis. IPF lung fibroblasts that were pretreated with GKT137831 were then treated with staurosporine to induce apoptosis. It was observed that when NOX4 activity was inhibited, fibroblasts were susceptible to apoptosis, compared to fibroblasts in which NOX4 function was intact. Taken together, these data indicate that NOX4 participates in inducing cellular senescence and resistance to apoptosis in IPF lung fibroblasts [104].

Hecker et al. also evaluated the antioxidant response by determining the expression of NRF2 in the lung tissue of patients with IPF. NRF2 expression was decreased in fibroblasts within fibroblastic foci generated in IPF. These data indicate that in IPF, an alteration occurs in cellular redox homeostasis, resulting in the elevated expression of the ROS-generating enzyme NOX4 and an altered capacity to induce NRF2 as an antioxidant response, which promotes the development of more severe fibrosis [104].

Furthermore, to confirm these findings, Hecker et al. evaluated the repair capacity in young 2-month-old mice and in elderly 18-month-old mice of the C57BL/6 strain, which were treated with bleomycin to induce pulmonary fibrosis. At 3 weeks and 4 months after bleomycin treatment, fibrosis resolution was assessed by Masson’s trichrome staining and a quantitative hydroxyproline assay. Aged mice did not demonstrate a decrease in total lung hydroxyproline and in collagen levels compared to young mice. Thus, the level of fibrosis did not show a significant difference among the analyzed groups. However, older mice showed a reduced ability to repair fibrosis compared to younger mice. Also, they observed that the lungs of elderly mice presented an accumulation of senescent myofibroblasts resistant to apoptosis due to an imbalance between NOX4 and NRF2, which coincides with data described previously [104].

#### 5.2.4. Murine Double Minute 4 (MDM4)

Myofibroblasts from IPF lungs showed resistance to apoptosis compared to fibroblasts from control subjects without IPF [105,106]. This is important in the pathophysiology of the disease, because for the resolution of fibrosis, the elimination of excess ECM and myofibroblasts is necessary for lung tissue regeneration to occur [107].

Murine double minute 4 (MDM4) is one of the main endogenous inhibitors of p53. MDM4 is largely localized to the cytoplasm. In response to genotoxic stimuli, MDM4 translocates to the nucleus where it binds to the transactivation domain of p53 and inhibits p53 target genes [108,109].

Qu et al. determined the role of MDM4 in the pathophysiology of IPF. They evaluated the expression levels of MDM4 in lung tissue from patients with IPF and in a murine model of fibrosis induced with bleomycin in aged mice (15 months) of the C57BL/6 strain. MDM4 was expressed at a higher rate in patients and mice with IPF compared to their controls. Furthermore, they cultured primary normal human lung fibroblasts in a polyacrylamide matrix with variable stiffness, from physiological to fibrotic lung stiffness. Matrix hardening increased ELK1-mediated MDM4 expression and decreased the level of active p53 without changing the expression of total p53. Therefore, MDM4 is an endogenous inhibitor of p53 regulated by matrix stiffness [110].

Reducing fibrotic lung matrix stiffness or the genetic depletion of MDM4 in lung myofibroblasts activates the MDM4-p53 pathway, resulting in the induction of apoptosis in myofibroblasts and promoting resolution of lung fibrosis in aged mice. These findings suggest that MDM4 is mechanosensitive; therefore, its expression is increased in fibrotic lungs, causing an inhibition of p53 activation that results in fibroblasts resistant to apoptosis and preventing the resolution of IPF [110].

These findings were confirmed in the study conducted by Mei et al., who used XI-011 (NSC149109), an MDM4 inhibitor, in a murine model of pulmonary fibrosis induced with bleomycin. They observed that treatment with XI-011 intratracheally had an anti-fibrotic effect by sensitizing lung myofibroblasts to apoptosis and promoting the elimination of these cells mediated by macrophages. Interestingly, they reported that XI-011 treatment caused minimal effect on the viability of normal lung fibroblasts and lung epithelial cells, while significantly activating apoptosis in lung myofibroblasts, which correspond to fibrotic cells. These results demonstrate that XI-011 may be a promising candidate for treating IPF. Despite the promising results, we consider that more studies are required before this treatment can be applied to humans [111].

### 5.3. Balance between Cell Proliferation and Apoptosis

#### 5.3.1. Ribose-5-Phosphate Isomerase A (RPIA)

As mentioned above, aging is associated with a higher incidence of cancer development [112]. Aging and cancer are related to metabolic dysregulation. Ribose-5-phosphate isomerase A (RPIA) is a key enzyme in the regulation of the non-oxidative pentose phosphate pathway (PPP). Activation of the PPP pathway has been suggested to enhance cancer cell growth [113]. It has been reported that RPIA expression is increased in several types of cancer including lung adenocarcinoma [114,115].

To evaluate the role of RPIA in tumor progression in the lung, Nieh et al. analyzed the effect of reducing the expression of this protein in A549 knockdown cells (*Rpia* KD). These cells showed reduced cell proliferation capacity through pERK/2 impairment and increased apoptosis rate by activating the intrinsic pathway. Furthermore, an increase in the expression of p53 and p21 was observed, inducing cellular senescence, autophagy, and the production of ROS and contributing to a reduction in cancer cell proliferation [115]. Together, these data show that RPIA causes an increase in cell proliferation causing it to predominate over apoptosis, which promotes tumor growth.

#### 5.3.2. Dehydroepiandrosterone (DHEA)

The adrenal steroid dehydroepiandrosterone (DHEA) and its hydrophilic storage form (DHEA-S) are the most abundant adrenal steroids in humans. Their levels reach their maximum concentration between the ages of 25 and 30 years and then decrease as age increases [116]. An abnormal decrease in the blood levels of DHEA has been associated with a deterioration of the immune system, similar to what occurs with aging. Additionally, low levels of DHEA are also associated with several chronic diseases [117].

Mendoza-Milla et al. examined the role of DHEA in IPF. They quantified the plasma levels of DHEA and DHEA-S in 137 patients with IPF and 58 controls, in an age range between 54 and 74 years. A decrease in DHEA and DHEA-S levels was observed in male patients, while only the DHEA levels decreased in female patients, compared to age-matched controls. They also evaluated the effect of DHEA on primary fibroblasts isolated from the lungs of IPF patients. When DHEA was added to the cell culture medium, a decrease in cell proliferation and an increase in apoptosis were observed due to an increase in the levels of the apoptosis receptors TNFR1 and TRAILR2 and the proapoptotic marker Bax and a decrease in the levels of the antiapoptotic proteins c-IAP1, c-IAP2, livin, survivin, and cercain. Importantly, DHEA treatment also caused the activation of caspase-9, indicating that DHEA induces apoptosis through the intrinsic pathway. These findings demonstrate that DHEA is related to the development of IPF because its decrease with age promotes the proliferation of fibroblasts and makes them resistant to apoptosis [118].

#### 5.3.3. Matrix Metalloproteinase 28 (MMP28)

Matrix metalloproteinases (MMPs) participate in the degradation of ECM components such as collagen, elastin, and casein, as well as of molecules involved in differentiation, proliferation, and angiogenesis [119]. As mentioned above, ECM dysregulation is a key feature of lung aging [26]. It has been suggested that abnormal MMP expression contributes to the pathogenesis and or progression of IPF [120].

Maldonado et al. evaluated MMP28 expression levels in tissue samples from IPF patients and healthy donors. They also evaluated the functional effects of MMP28 in primary cultures of AEC-II and basal BECs isolated from IPF patients and healthy donors. The age range of the people who participated in this study was 34–62 years. They found that MMP28 expression was increased in patients with IPF, showing greater expression in the cytoplasm and nucleus of AEC-II and in the apical region and cytoplasm of basal BECs, compared to the control group [121].

In vitro, AECs overexpressing MMP28 showed increased cell proliferation and protection against bleomycin-induced apoptosis. In contrast, silencing of MMP28 decreased the cell proliferation rates and increased apoptosis. Basal BECs showed a similar response. To further explore the role of MMP28 in the development of IPF, they silenced MMP28 in a murine model of fibrosis in the C57BL/6 strain and found that after 14 days of bleomycin treatment, MMP28-deficient mice developed less fibrosis in the lung [121].

These findings demonstrate that MMP28 plays a role in the aberrant epithelial cell phenotype that characterizes IPF.

The data analyzed above indicate that an imbalance between cell proliferation and apoptosis triggers the development of age-related lung diseases. A balance must be maintained between these processes for adequate cell replacement to occur.

Table 1 summarizes the effects caused in lung aging by the regulatory molecules of cell proliferation and apoptosis analyzed in this review.

## 6. Conclusions

Lung changes associated with aging contribute to older people’s increased susceptibility to serious respiratory diseases. In this work, we analyzed articles that evaluated the role of molecules involved in cell proliferation and apoptosis in lung aging and in the development of age-related lung diseases.

Some molecules such as TNC, FOXM1, DNA-PKcs, and microRNAs regulate the cell proliferation process, others modify apoptosis, such as BCL-W, BCL-XL, TCF21, p16, NOX4, NRF2, and MDM4, while there are molecules such as RPIA, DHEA, and MMP28 that intervene in the balance between both phenomena.

The main lung tissue cells affected by changes in cellular turnover during the normal aging process are BECs, AEC-I, AEC-II, and fibroblasts.

However, despite the studies carried out to date, the complete signaling pathways that regulate cell turnover in lung aging are still unknown. The use of appropriate experimental methodologies in adequate in vivo and in vitro models will help to elucidate those pathways in the future [122,123].

Studying the aging process in the lung and its related diseases could allow for the design of preventive, diagnostics, and therapeutic strategies, which is of utmost importance considering that life expectancy and the number of older people continue to increase worldwide.

## Figures and Tables

**Table 1:** Molecules that regulate cell proliferation and apoptosis during lung aging.

Molecule	Model	Effect	Reference
TNC	*Tnc* KO mice of strain 129/Sv	TNC deficiency increased cell proliferation in the lung parenchyma	Gremlich et al. (2021) [78]
FOXM1	Inflammatory lung injury in aged mice of the strain C57/BL6	FOXM1 deficiency decreased cell proliferation in lung endothelial cells	Huang et al. (2024) [86]
DNA-PKcs	Primary lung fibroblasts	DNA-PKcs deficiency promoted the proliferation of SSEA4+ mesenchymal progenitor cells and myofibroblasts	Habiel et al. (2019) [91]
MicroRNAs	Senescent human fibroblasts	Elevated MicroRNA expression caused cell cycle arrest	Markopoulos et al. (2017) [95] Maes et al. (2009) [96]
miR-34a	Model of fibrosis in aged *miR-34a* KO mice	miR-34a deficiency increased cell proliferation and prevented the development of fibrosis	Cui et al. (2017) [97]
BCL-W and BCL-XL	Induced senescent IMR-90 cells	Elevated BCL-W and BCL-XL expression caused resistance to apoptosis	Yosef et al. (2016) [98]
TCF21	SCID mice injected with p16-deficient lung cancer cells	TCF21 activation induced caspase-3-dependent apoptosis in response to telomere damage	Selvam et al. (2018) [101]
p16	SCID mice	p16 activation induced cellular senescence with resistance to apoptosis in response to telomere damage	Selvam et al. (2018) [101]
NOX4	Fibroblasts from lungs of patients with IPF	Elevated NOX4 expression promoted cellular senescence and resistance to apoptosis	Hecker et al. (2014) [104]
	Aged C57BL/6 strain mice	Elevated NOX-4 expression decreased lung regeneration capacity	
NRF2	Fibroblasts from lungs of patients with IPF	Nrf2 deficiency promoted cellular senescence and resistance to apoptosis	Hecker et al. (2014) [104]
	Aged C57BL/6 strain mice	Nrf2 deficiency decreased lung regeneration capacity	
MDM4	Model of fibrosis in the C57BL/6 strain	Elevated MDM4 expression caused resistance to apoptosis	Qu et al. (2021) [110]
RPIA	A549 *Rpia* KD cells	RPIA deficiency caused a decrease in proliferation and an increase in apoptosis	Nieh et al. (2022) [115]
DHEA	Fibroblasts from lungs of patients with IPF	DHEA deficiency promoted cell proliferation and resistance against apoptosis	Mendoza-Milla et al. (2013) [118]
MMP28	Primary cultures of AEC-II and basal BECs from IPF patients	Elevated MMP28 expression showed increased cell proliferation and protection against apoptosis	Maldonado et al. (2018) [121]

TNC: tenascin-C; KO: knockout; FOXM1: forkhead box M1; DNA-PKcs: DNA protein kinase catalytic subunit; SSEA4+: stage-specific embryonic antigen-4; BCL-W: Bcl-2-like protein 2; BL-XL: B-cell lymphoma-extra-large; TCF21: transcription factor 21; SCID mice: severe combined immunodeficient mice; p16: cyclin-dependent kinase inhibitor 2A; NOX-4: NADPH oxidase-4; Nrf2: NFE2-related factor 2; RPIA: ribose-5-phosphate isomerase A; KD: knockdown; DHEA: dehydroepiandrosterone; MMP28: matrix metalloproteinase 28; AEC-II: alveolar epithelial cells type II; BECs: bronchial epithelial cells.

## Data Availability

Data sharing is not applicable to this review paper.

## References

[1] López-Otín C., Blasco M.A., Partridge L., Serrano M., Kroemer G. (2013). The hallmarks of aging. Cell.

[2] A World Population Prospects 2022. https://population.un.org/wpp/Graphs/Probabilistic/PopPerc/65plus/900.

[3] Budinger G.R.S., Kohanski R.A., Gan W., Kobor M.S., Amaral L.A., Armanios M., Kelsey K.T., Pardo A., Tuder R., Macian F. (2017). The Intersection of Aging Biology and the Pathobiology of Lung Diseases: A Joint NHLBI/NIA Workshop. J. Gerontol. A Biol. Sci. Med. Sci..

[4] Life Expectancy and Healthy Life Expectancy. https://apps.who.int/gho/data/view.main.SDG2016LEXREGv?lang=en.

[5] Niccoli T., Partridge L. (2012). Ageing as a risk factor for disease. Curr. Biol..

[6] Schneider J.L., Rowe J.H., Garcia-de-Alba C., Kim C.F., Sharpe A.H., Haigis M.C. (2021). The aging lung: Physiology, disease, and immunity. Cell.

[7] Sharma G., Goodwin J. (2006). Effect of aging on respiratory system physiology and immunology. Clin. Interv. Aging.

[8] Wang L., Green F.H.Y., Smiley-Jewell S.M., Pinkerton K.E. (2010). Susceptibility of the aging lung to environmental injury. Semin. Respir. Crit. Care Med..

[9] Quirk J.D., Sukstanskii A.L., Woods J.C., Lutey B.A., Conradi M.S., Gierada D.S., Yusen R.D., Castro M., Yablonskiy A.A. (2016). Experimental evidence of age-related adaptive changes in human acinar airways. J. Appl. Physiol..

[10] Brandsma C.A., de Vries M., Costa R., Woldhuis R.R., Konigshoff M., Timens W. (2017). Lung ageing and COPD: Is there a role for ageing in abnormal tissue repair?. Eur. Respir. Rev..

[11] Jaramillo-Rangel G., Gutiérrez-Arenas E., Ancer-Arellano A., Chávez-Briones M.L., Cerda-Flores R.M., Ortega-Martínez M., Gul O. (2022). Determination of the area and number of pulmonary alveoli through the normal aging process in CD1 mouse. Research Aspects in Biological Science.

[12] Ortega-Martínez M., Gopar-Cuevas Y., Chavez-Briones M.L., Miranda-Maldonado I., Ancer-Arellano A., Alvarez-Cuevas S., Solís-Soto J.M., Ancer-Rodriguez J., Jaramillo-Rangel G., Ghannam W.M. (2024). Evaluation of the Number of Alveolar Attachments through the Normal Aging Process in the Lung of CD1 Mouse. New Visions in Medicine and Medical Science.

[13] Cho S.J., Stout-Delgado H.W. (2020). Aging and Lung Disease. Annu. Rev. Physiol..

[14] Childs B.G., Durik M., Baker D.J., van Deursen J.M. (2015). Cellular senescence in aging and age-related disease: From mechanisms to therapy. Nat. Med..

[15] Szalontai K., Gémes N., Furák J., Varga T., Neuperger P., Balog J.Á., Puskás L.G., Szebeni G.J. (2021). Chronic Obstructive Pulmonary Disease: Epidemiology, Biomarkers, and Paving the Way to Lung Cancer. J. Clin. Med..

[16] Burney P., Jarvis D., Perez-Padilla R. (2015). The global burden of chronic respiratory disease in adults. Int. J. Tuberc. Lung Dis..

[17] Bos L.D.J., Ware L.B. (2022). Acute respiratory distress syndrome: Causes, pathophysiology, and phenotypes. Lancet.

[18] Matthay M.A., Zemans R.L., Zimmerman G.A., Arabi Y.M., Beitler J.R., Mercat A., Herridge M., Randolph A.G., Calfee C.S. (2019). Acute respiratory distress syndrome. Nat. Rev. Dis. Primers.

[19] Angus D.C., Linde-Zwirble W.T., Lidicker J., Clermont G., Carcillo J., Pinsky M.R. (2001). Epidemiology of severe sepsis in the United States: Analysis of incidence, outcome, and associated costs of care. Crit. Care Med..

[20] Sloane P.J., Gee M.H., Gottlieb J.E., Albertine K.H., Peters S.P., Burns J.R., Machiedo G., Fis J.E. (1992). A multicenter registry of patients with acute respiratory distress syndrome. Physiology and outcome. Am. Rev. Respir. Dis..

[21] Hattab Y., Alhassan S., Balaan M., Lega M., Singh A.C. (2016). Chronic Obstructive Pulmonary Disease. Crit. Care Nurs. Q..

[22] Brusasco V., Martinez F. (2014). Chronic obstructive pulmonary disease. Compr. Physiol..

[23] Kahnert K., Jörres R.A., Behr J., Welte T. (2023). The Diagnosis and Treatment of COPD and Its Comorbidities. Dtsch. Arztebl. Int..

[24] Rabe K.F., Watz H. (2017). Chronic obstructive pulmonary disease. Lancet.

[25] Wachami N., Guennouni M., Iderdar Y., Boumendil K., Arraji M., Mourajid Y., Bouchachi F.Z., Barkaoui M., Louerdi M.L., Hilali A. (2024). Estimating the global prevalence of chronic obstructive pulmonary disease (COPD): A systematic review and meta-analysis. BMC Public Health.

[26] Meiners S., Eickelberg O., Königshoff M. (2015). Hallmarks of the ageing lung. Eur. Respir. J..

[27] Martinez F.J., Collard H.R., Pardo A., Raghu G., Richeldi L., Selman M., Swigris J.J., Taniguchi H., Wells A.U. (2017). Idiopathic pulmonary fibrosis. Nat. Rev. Dis. Primers.

[28] Spagnolo P., Kropski J.A., Jones M.G., Lee J.S., Rossi G., Karampitsakos T., Maher T.M., Tzouvelekis A., Ryerson C.R. (2021). Idiopathic pulmonary fibrosis: Disease mechanisms and drug development. Pharmacol. Ther..

[29] Glass D.S., Grossfeld D., Renna H.A., Agarwala P., Spiegler P., DeLeon J., Reiss A.B. (2022). Idiopathic pulmonary fibrosis: Current and future treatment. Clin. Respir. J..

[30] Pergolizzi J.V., LeQuang J.A., Varrassi M., Breve F., Magnusson P., Varrassi G. (2023). What Do We Need to Know About Rising Rates of Idiopathic Pulmonary Fibrosis? A Narrative Review and Update. Adv. Ther..

[31] Li X., Wang Y., Liang J., Bi Z., Ruan H., Cui Y., Ma L., Wei Y., Zhou B., Zhang L. (2021). Bergenin attenuates bleomycin-induced pulmonary fibrosis in mice via inhibiting TGF-beta1 signaling pathway. Phytother. Res..

[32] Sacco P.C., Casaluce F., Sgambato A., Rossi A., Maione P., Palazzolo G., Napolitano A., Gridelli C. (2015). Current challenges of lung cancer care in an aging population. Expert. Rev. Anticancer Ther..

[33] Bray F., Laversanne M., Sung H., Ferlay J., Siegel R.L., Soerjomataram I., Jemal A. (2024). Global cancer statistics 2022: GLOBOCAN estimates of incidence and mortality worldwide for 36 cancers in 185 countries. CA Cancer J. Clin..

[34] Gridelli C., Langer C., Maione P., Rossi A., Schild S.E. (2007). Lung cancer in the elderly. J. Clin. Oncol..

[35] Campisi J. (2016). Cellular Senescence and Lung Function during Aging. Yin and Yang. Ann. Am. Thorac. Soc..

[36] Wang C., Jurk D., Maddick M., Nelson G., Martin-Ruiz C., Von Zglinicki T. (2009). DNA damage response and cellular senescence in tissues of aging mice. Aging Cell.

[37] Chilosi M., Carloni A., Rossi A., Poletti V. (2013). Premature lung aging and cellular senescence in the pathogenesis of idiopathic pulmonary fibrosis and COPD/emphysema. Transl. Res..

[38] Karrasch S., Holz O., Jörres R.A. (2008). Aging and induced senescence as factors in the pathogenesis of lung emphysema. Respir. Med..

[39] Navarro S., Driscoll B. (2017). Regeneration of the Aging Lung: A Mini-Review. Gerontology.

[40] Medh R.D., Thompson E.B. (2000). Hormonal regulation of physiological cell turnover and apoptosis. Cell Tissue Res..

[41] Aoshiba K., Nagai A. (2009). Senescence hypothesis for the pathogenetic mechanism of chronic obstructive pulmonary disease. Proc. Am. Thorac. Soc..

[42] Dang C., Gilewski T.A., Surbone A., Norton L., Kufe D.W., Pollock R.E., Weichselbaum R.R., Bast R.C., Gansler T.S., Holland J.F., Frei E. (2003). Cell Proliferation. Cancer Medicine.

[43] Elmore S. (2007). Apoptosis: A review of programmed cell death. Toxicol. Pathol..

[44] Igney F.H., Krammer P.H. (2002). Death and anti-death: Tumour resistance to apoptosis. Nat. Rev. Cancer.

[45] Sender R., Milo R. (2021). The distribution of cellular turnover in the human body. Nat. Med..

[46] Wansleeben C., Bowie E., Hotten D.F., Yu Y.R., Hogan B.L. (2014). Age-related changes in the cellular composition and epithelial organization of the mouse trachea. PLoS ONE.

[47] Walski M., Pokorski M., Antosiewicz J., Rekawek A., Frontczak-Baniewicz M., Jernajczyk U., Di Giulio C. (2009). Pulmonary surfactant: Ultrastructural features and putative mechanisms of aging. J. Physiol. Pharmacol..

[48] Hong K.U., Reynolds S.D., Watkins S., Fuchs E., Stripp B.R. (2004). In vivo differentiation potential of tracheal basal cells: Evidence for multipotent and unipotent subpopulations. Am. J. Physiol.-Lung Cell. Mol. Physiol..

[49] Ortega-Martínez M., Rodríguez-Flores L.E., Ancer-Arellano A., Cerda-Flores R.M., de-la-Garza-González C., Ancer-Rodríguez J., Jaramillo-Rangel G. (2016). Analysis of Cell Turnover in the Bronchiolar Epithelium through the Normal Aging Process. Lung.

[50] Angelidis I., Simon L.M., Fernandez I.E., Strunz M., Mayr C.H., Greiffo F.R., Tsitsiridis G., Ansari M., Graf E., Strom T.M. (2019). An atlas of the aging lung mapped by single cell transcriptomics and deep tissue proteomics. Nat. Commun..

[51] Borok Z., Horie M., Flodby P., Wang H., Liu Y., Ganesh S., Firth A.L., Minoo P., Li C., Beers M.F. (2020). Grp78 Loss in Epithelial Progenitors Reveals an Age-linked Role for Endoplasmic Reticulum Stress in Pulmonary Fibrosis. Am. J. Respir. Crit. Care Med..

[52] Nabhan A.N., Brownfield D.G., Harbury P.B., Krasnow M.A., Desai T.J. (2018). Single-cell Wnt signaling niches maintain stemness of alveolar type 2 cells. Science.

[53] Del Riccio V., van Tuyl M., Post M. (2004). Apoptosis in lung development and neonatal lung injury. Pediatr. Res..

[54] Burri P.H., Moschopulos M. (1992). Structural analysis of fetal rat lung development. Anat. Rec..

[55] De Paepe M.E., Rubin L.P., Jude C., Lesieur-Brooks A.M., Mills D.R., Luks F.I. (2000). Fas ligand expression coincides with alveolar cell apoptosis in late-gestation fetal lung development. Am. J. Physiol.-Lung Cell. Mol. Physiol..

[56] Nagata S., Golstein P. (1995). The Fas death factor. Science.

[57] Nagata S. (1997). Apoptosis by death factor. Cell.

[58] Volpe E., Sambucci M., Battistini L., Borsellino G. (2016). Fas-Fas ligand: Checkpoint of T cell functions in multiple sclerosis. Front. Immunol..

[59] De Paepe M.E., Mao Q., Embree-Ku M., Rubin L.P., Luks F.I. (2004). Fas/FasL-mediated apoptosis in perinatal murine lungs. Am. J. Physiol.-Lung Cell. Mol. Physiol..

[60] Yoshimi M., Maeyama T., Yamada M., Hamada N., Fukumoto J., Kawaguchi T., Kuwano K., Nakanishi Y. (2008). Recombinant human erythropoietin reduces epithelial cell apoptosis and attenuates bleomycin-induced pneumonitis in mice. Respirology.

[61] Foster D.J., Moe O.W., Hsia C.C. (2004). Upregulation of erythropoietin receptor during postnatal and postpneumonectomy lung growth. Am. J. Physiol.-Lung Cell. Mol. Physiol..

[62] Paxson J.A., Gruntman A., Parkin C.D., Mazan M.R., Davis A., Ingenito E.P., Hoffman A.M. (2011). Age-dependent decline in mouse lung regeneration with loss of lung fibroblast clonogenicity and increased myofibroblastic differentiation. PLoS ONE.

[63] Chiquet-Ehrismann R., Mackie E.J., Pearson C.A., Sakakura T. (1986). Tenascin: An extracellular matrix protein involved in tissue interactions during fetal development and oncogenesis. Cell.

[64] Lambropoulou M., Limberis V., Koutlaki N., Simopoulou M., Ntanovasilis D., Vandoros G.P., Tatsidou P., Kekou I., Koutsikogianni I., Papadopoulos N. (2009). Differential expression of tenascin-C in the developing human lung: An immunohistochemical study. Clin. Exp. Med..

[65] Mund S.I., Schittny J.C. (2020). Tenascin-C deficiency impairs alveolarization and microvascular maturation during postnatal lung development. J. Appl. Physiol..

[66] Schmidt A.M., Yan S.D., Yan S.F., Stern D.M. (2001). The multiligand receptor RAGE as a progression factor amplifying immune and inflammatory responses. J. Clin. Investig..

[67] Reynolds P.R., Kasteler S.D., Cosio M.G., Sturrock A., Huecksteadt T., Hoidal R.A. (2008). Developmental expression and positive feedback regulation by EGR-1 during cigarette smoke exposure in pulmonary epithelial cells. Am. J. Physiol.-Lung Cell. Mol. Physiol..

[68] Sharma A., Kaur S., Sarkar M., Sarin B.C., Changotra H. (2021). The AGE-RAGE Axis and RAGE Genetics in Chronic Obstructive Pulmonary Disease. Clin. Rev. Allergy Immunol..

[69] Dahlin K., Mager E.M., Allen L., Tigue Z., Goodglick L., Wadehra M., Dobbs L. (2004). Identification of genes differentially expressed in rat alveolar type I cells. Am. J. Respir. Cell Mol. Biol..

[70] Blondonnet R., Audard J., Belville C., Clairefond G., Lutz J., Bouvier D., Roszyk L., Gross C., Lavergne M., Fournet M. (2017). RAGE inhibition reduces acute lung injury in mice. Sci. Rep..

[71] Fineschi S., De Cunto G., Facchinetti F., Civelli M., Imbimbo B.P., Carnini C., Villetti G., Lunghi B., Stochino S., Gibbons D.L. (2013). Receptor for advanced glycation end products contributes to postnatal pulmonary development and adult lung maintenance program in mice. Am. J. Respir. Cell Mol. Biol..

[72] Go H., Ohto H., Nollet K.E., Sato K., Miyazaki K., Maeda H., Ichikawa H., Chishiki M., Kashiwabara N., Kume Y. (2021). Biomarker Potential of the Soluble Receptor for Advanced Glycation End Products to Predict Bronchopulmonary Dysplasia in Premature Newborns. Front. Pediatr..

[73] Guo W.A., Knight P.R., Raghavendran K. (2012). The receptor for advanced glycation end products and acute lung injury/acute respiratory distress syndrome. Intensive Care Med..

[74] Englert J.M., Hanford L.E., Kaminski N., Tobolewski J.M., Tan R.J., Fattman C.L., Ramsgaard L., Richards T.J., Loutaev I., Nawroth P.P. (2008). A role for the receptor for advanced glycation end products in idiopathic pulmonary fibrosis. Am. J. Pathol..

[75] Donovan C., Bai X., Chan Y.L., Feng M., Ho K., Guo H., Chen H., Oliver B.G. (2023). Tenascin C in lung diseases. Biology.

[76] Jones F.S., Jones P.L. (2000). The tenascin family of ECM glycoproteins: Structure, function, and regulation during embryonic development and tissue remodeling. Dev. Dyn..

[77] Löfdah M., Kaarteenaho R., Lappi-Blanco E., Tornling G., Sköld M.C. (2011). Tenascin-C and alpha-smooth muscle actin positive cells are increased in the large airways in patients with COPD. Respir. Res..

[78] Gremlich S., Cremona T.P., Yao E., Chabenet F., Fytianos K., Roth-Kleiner M., Schittny J.C. (2021). Tenascin-C: Friend or foe in lung aging?. Front. Physiol..

[79] Chen X., Müller G.A., Quaas M., Fischer M., Han N., Stutchbury B., Sharrocks A.D., Engeland K. (2013). The forkhead transcription factor FOXM1 controls cell cycle-dependent gene expression through an atypical chromatin binding mechanism. Mol. Cell. Biol..

[80] Smirnov A., Panatta E., Lena A.M., Castiglia D., Di Daniele N., Gerry Melino G., Candi E. (2016). FOXM1 regulates proliferation, senescence and oxidative stress in keratinocytes and cancer cells. Aging.

[81] Ribeiro R., Macedo J.C., Costa M., Ustiyan V., Shindyapina A.V., Tyshkovskiy A., Gomes R.N., Castro J.P., Kalin T.V., Vasques-Nóvoa F. (2022). In vivo cyclic induction of the FOXM1 transcription factor delays natural and progeroid aging phenotypes and extends healthspan. Nat. Aging.

[82] Zhao Y., Gao X., Zhao Y.D., Mirza M.K., Frey R.S., Kalinichenko V.V., Wang I., Costa R.H., Malik A.B. (2006). Endothelial cell-restricted disruption of FoxM1 impairs endothelial repair following LPS-induced vascular injury. J. Clin. Investig..

[83] Zhao Y.D., Huang X., Yi F., Dai Z., Qian Z., Tiruppathi C., Tran K., Zhao Y. (2014). Endothelial FoxM1 mediates bone marrow progenitor cell-induced vascular repair and resolution of inflammation following inflammatory lung injury. Stem Cells.

[84] Aird W.C. (2003). The role of the endothelium in severe sepsis and multiple organ dysfunction syndrome. Blood.

[85] Goldenberg N.M., Steinberg B.E., Slutsky A.S., Lee W.L. (2011). Broken barriers: A new take on sepsis pathogenesis. Sci. Transl. Med..

[86] Huang X., Zhang X., Machireddy N., Evans C.E., Trewartha S.D., Hu G., Fang Y., Mutlu G.M., Wu D., Zhao Y. (2023). Endothelial FoxM1 reactivates aging-impaired endothelial regeneration for vascular repair and resolution of inflammatory lung injury. Sci. Transl. Med..

[87] Zhou S.Y., Lee J., Jiang W., Crowe J.L., Zha S., Paull T.T. (2017). Regulation of the DNA damage response by DNA-PKcs inhibitory phosphorylation of ATM. Mol. Cell.

[88] Yue X., Bai C., Xie D., Ma T., Zhou P. (2020). DNA-PKcs: A multi-faceted player in DNA damage response. Front. Genet..

[89] Kumar V., Fleming T., Terjung S., Gorzelanny C., Gebhardt C., Agrawal R., Mall M.A., Ranzinger J., Zeier M., Madhusudhan T. (2017). Homeostatic nuclear RAGE-ATM interaction is essential for efficient DNA repair. Nucleic Acids Res..

[90] Habiel D.M., Camelo A., Espindola M., Burwell T., Hanna R., Miranda E., Carruthers A., Bell M., Coelho A.L., Liu H. (2017). Divergent roles for clusterin in lung injury and repair. Sci. Rep..

[91] Habiel D.M., Hohmann M.S., Espindola M.S., Coelho A.L., Jones I., Jones H., Carnibella R., Pinar I., Werdiger F., Hogaboam C.M. (2019). DNA-PKcs modulates progenitor cell proliferation and fibroblast senescence in idiopathic pulmonary fibrosis. BMC Pulm. Med..

[92] Xia H., Bodempudi V., Benyumov A., Hergert P., Tank D., Herrera J., Braziunas J., Larsson O., Parker M., Rossi D. (2014). Identification of a cell-of-origin for fibroblasts comprising the fibrotic reticulum in idiopathic pulmonary fibrosis. Am. J. Pathol..

[93] Saliminejad K., Khorshid H.R.K., Fard S.S., Ghaffari S.H. (2019). An overview of microRNAs: Biology, functions, therapeutics, and analysis methods. J. Cell. Physiol..

[94] Ranganathan K., Sivasankar V. (2014). MicroRNAs—Biology and clinical applications. J. Oral Maxillofac. Pathol..

[95] Markopoulos S.G., Roupakia E., Tokamani M., Vartholomatos G., Tzavaras T., Hatziapostolou M., Fackelmayer F.O., Sandaltzopoulos R., Polytarchou C., Kolettas E. (2017). Senescence-associated microRNAs target cell cycle regulatory genes in normal human lung fibroblasts. Exp. Gerontol..

[96] Maes O.C., Sarojini H., Wang E. (2009). Stepwise up-regulation of microRNA expression levels from replicating to reversible and irreversible growth arrest states in WI-38 human fibroblasts. J. Cell. Physiol..

[97] Cui H., Ge J., Xie N., Banerjee S., Zhou Y., Liu R.M., Thannickal V.J., Liu G. (2017). miR-34a promotes fibrosis in aged lungs by inducing alveolar epithelial dysfunctions. Am. J. Physiol.-Lung Cell. Mol. Physiol..

[98] Yosef R., Pilpel N., Tokarsky-Amiel R., Biran A., Ovadya Y., Cohen S., Vadai E., Dassa L., Shahar E., Condiotti R. (2016). Directed elimination of senescent cells by inhibition of BCL-W and BCL-XL. Nat. Commun..

[99] Zvereva M.I., Shcherbakova D.M., Dontsova O.A. (2010). Telomerase: Structure, functions, and activity regulation. Biochemistry.

[100] Shay J.W. (2016). Role of telomeres and telomerase in aging and cancer. Cancer Discov..

[101] Selvam S.P., Roth B.M., Nganga R., Kim J., Cooley M.A., Helke K., Smith C.D., Ogretmen B. (2018). Balance between senescence and apoptosis is regulated by telomere damage-induced association between p16 and caspase-3. J. Biol. Chem..

[102] Guo S., Chen X. (2015). The human Nox4: Gene, structure, physiological function and pathological significance. J. Drug Target..

[103] Ma Q. (2013). Role of nrf2 in oxidative stress and toxicity. Annu. Rev. Pharmacol. Toxicol..

[104] Hecker L., Logsdon N.J., Kurundkar D., Kurundkar A., Bernard K., Hock T., Meldrum E., Sanders Y.Y., Thannickal V.J. (2014). Reversal of persistent fibrosis in aging by targeting Nox4-Nrf2 redox imbalance. Sci. Transl. Med..

[105] Akram K.M., Lomas N.J., Forsyth N.R., Spiteri M.A. (2014). Alveolar epithelial cells in idiopathic pulmonary fibrosis display upregulation of TRAIL, DR4 and DR5 expression with simultaneous preferential over-expression of pro-apoptotic marker p53. Int. J. Clin. Exp. Pathol..

[106] Cisneros J., Hagood J., Checa M., Ortiz-Quintero B., Negreros M., Herrera I., Ramos C., Pardo A., Selman M. (2012). Hypermethylation-mediated silencing of p14(ARF) in fibroblasts from idiopathic pulmonary fibrosis. Am. J. Physiol.-Lung Cell. Mol. Physiol..

[107] Desmoulière A., Redard M., Darby I., Gabbiani G. (1995). Apoptosis mediates the decrease in cellularity during the transition between granulation tissue and scar. Am. J. Pathol..

[108] Shvarts A., Steegenga W.T., Riteco N., van Laar T., Dekker P., Bazuine M., van Ham R.C., van der Houven van Oordt W., Hateboer G., van der Eb A.J. (1996). MDMX: A novel p53-binding protein with some functional properties of MDM2. EMBO J..

[109] Finch R.A., Donoviel D.B., Potter D., Shi M., Fan A., Freed D.D., Wang C., Zambrowicz B.P., Ramirez-Solis R., Sands A.T. (2002). mdmx is a negative regulator of p53 activity in vivo. Cancer Res..

[110] Qu J., Yang S., Zhu Y., Guo T., Thannickal V.J., Zhou Y. (2021). Targeting mechanosensitive MDM4 promotes lung fibrosis resolution in aged mice. J. Exp. Med..

[111] Mei Q., Yang Z., Xiang Z., Zuo H., Zhou Z., Dong X., Zhang L., Song W., Wang Y., Hu Q. (2023). Pharmacological inhibition of MDM4 alleviates pulmonary fibrosis. Theranostics.

[112] Campisi J. (2013). Aging, cellular senescence, and cancer. Annu. Rev. Physiol..

[113] Du W., Jiang P., Mancuso A., Stonestrom A., Brewer M.D., Minn A.J., Mak T.W., Wu M., Yang X. (2013). TAp73 enhances the pentose phosphate pathway and supports cell proliferation. Nat. Cell Biol..

[114] Spencer N., Hopkinson D.A. (1980). Biochemical genetics of the pentose phosphate cycle: Human ribose 5-phosphate isomerase (RPI) and ribulose 5-phosphate 3-epimerase (RPE). Ann. Hum. Genet..

[115] Nieh Y.C., Chou Y.T., Chou Y.T., Wang C.Y., Lin S.X., Ciou S.C., Yuh C.H., Wang H.D. (2022). Suppression of ribose-5-phosphate isomerase a induces ROS to activate autophagy, apoptosis, and cellular senescence in lung cancer. Int. J. Mol. Sci..

[116] Makrantonaki E., Schonknecht P., Hossini A.M., Kaiser E., Katsouli M.M., Adjaye J., Schröder J., Zouboulis C.C. (2010). Skin and brain age together: The role of hormones in the ageing process. Exp. Gerontol..

[117] Buford T.W., Willoughby D.S. (2008). Impact of DHEA(S) and cortisol on immune function in aging: A brief review. Appl. Physiol. Nutr. Metab..

[118] Mendoza-Milla C., Valero-Jiménez A., Rangel C., Lozano A., Morales V., Becerril C., Chavira R., Ruiz V., Barrera L., Montaño M. (2013). Dehydroepiandrosterone has strong antifibrotic effects and is decreased in idiopathic pulmonary fibrosis. Eur. Respir. J..

[119] Pardo A., Cabrera S., Maldonado M., Selman M. (2016). Role of matrix metalloproteinases in the pathogenesis of idiopathic pulmonary fibrosis. Respir. Res..

[120] Bonnans C., Chou J., Werb Z. (2014). Remodelling the extracellular matrix in development and disease. Nat. Rev. Mol. Cell Biol..

[121] Maldonado M., Salgado-Aguayo A., Herrera I., Cabrera S., Ortíz-Quintero B., Staab-Weijnitz C.A., Eickelberg O., Ramírez R., Manicone A.M., Selman M. (2018). Upregulation and Nuclear Location of MMP28 in Alveolar Epithelium of Idiopathic Pulmonary Fibrosis. Am. J. Respir. Cell Mol. Biol..

[122] Jaramillo-Rangel G., Chávez-Briones M.D., Ancer-Arellano A., Miranda-Maldonado I., Ortega-Martínez M. (2023). Back to the Basics: Usefulness of Naturally Aged Mouse Models and Immunohistochemical and Quantitative Morphologic Methods in Studying Mechanisms of Lung Aging and Associated Diseases. Biomedicines.

[123] D’Adamo G.L., Widdop J.T., Giles E.M. (2021). The future is now? Clinical and translational aspects of “Omics” technologies. Immunol. Cell Biol..

